# Digital twins for managing railway maintenance and resilience

**DOI:** 10.12688/openreseurope.13806.1

**Published:** 2021-08-04

**Authors:** Sakdirat Kaewunruen, Jessada Sresakoolchai, Yi-hsuan Lin

**Affiliations:** 1 Civil Engineering, University of Birmingham, Birmingham, B15 2TT, UK

**Keywords:** Building Information Modeling Railway Maintenance, Resilience, Digital Twin, Railway

## Abstract

**Background: **To improve railway construction and maintenance, a novel digital twin that helps stakeholders visualize, share data, and monitor the progress and the condition during services is required. Building Information Modelling (BIM) is a digitalization tool, which adopts an interoperable concept that benefits the whole life-cycle assessment (LCA) of the project. BIM’s applications create higher performance on cost efficiency and optimal time schedule, helping to reduce any unexpected consumption and waste over the life cycle of the infrastructure.

**Methods: **The digital twin will be developed using BIM embedded by the lifecycle analysis method. A case study based on Taipei Metro (TM) has been conducted to enhance the performance in operation and maintenance. Life cycles of TM will be assessed and complied with ISO14064. Operation and maintenance activities will be determined from official records provided by TM. Material flows, stocks, and potential risks in the LCA are analyzed using BIM quantification embedded by risk data layer obtained from TM. Greenhouse emission, cost consumption and expenditure will be considered for integration into the BIM.

**Results: **BIM demonstrated strong potential to enable a digital twin for managing railway maintenance and resilience. Based on the case study, a key challenge for BIM in Taiwan is the lack of insights, essential data, and construction standards, and thus the practical adoption of BIM for railway maintenance and resilience management is still in the design phase.

**Conclusions: **This study exhibits a practical paradigm of the digital twin for railway maintenance and resilience improvement. It will assist all stakeholders to engage in the design, construction, and maintenance enhancing the reduction in life cycle cost, energy consumption and carbon footprint. New insight based on the Taipei Mass Rapid Transit system is highly valuable for railway industry globally by increasing the lifecycle sustainability and improving resilience of railway systems.

## Introduction

Rapid developments in railway transportation infrastructures enhance the congestion and complexities of the construction so that it becomes difficult to manage the works which consist of many tasks, activities, and components. In addition, the public demand for the improvement of life quality in the metropolitan results in the revolution in railway constructions. In Taiwan, the public has a high dependence on transportation by MRT (Taipei Mass Rapid Transit) in Taipei city. Department of Rapid Transit Systems declared that the average of the annual transport volume in January had greatly increased from 145.45 million to 194.63 million passengers in eight years (2012–2019), which corresponds to an annual average growth rate of approximately 4%.

Building Information Modelling (BIM) is a process to digitise and simulate the built environments that is beneficial to project management, monitoring and operation during the whole life-cycle assessment (LCA) progress in real-time. Relevant parties can access the BIM database to use the information. A study that collected reports from 92 random organizations, stated that in the UK at present, approximately 54.88% of organizations adopt BIM in the design stage for data sharing among stakeholders. The achievement in stakeholder collaboration by BIM is the most significant advantage to firms ^1^. Becerik-Gerber *et al*. ^2^ declared that the consumption of energy, capital, and so on in the maintenance stage are much higher than other stages in the whole life cycle of projects. Therefore, further BIM application for the operation and maintenance phases is necessary. In practice, residual risks and uncertainty (e.g. handover defects, natural hazards, etc.) influence the maintainability of infrastructure systems after construction phase, especially in railway systems, since any omissions of the risk management would increase the possibility of accidents, and can lead to serious casualties. The train route between Chiang Kai-shek Memorial Hall Station and Dongmen Station is used here as a case study to demonstrate the extended application of BIM for LCA of the Mass Rapid Transit (MRT) railway infrastructures. The BIM has been embedded with multiple data layers (capable of near real-time updating) to generate a digital twin of the MRT section. As many routes of the MRT are underground tunnels with complex pipelines, operational safety is vital. Railways in underground tunnels can be affected or dislocated by surrounding environmental factors such as earthquakes, floods, etc. This study aims to assess the MRT system using the digital twin to enhance the sustainability of the construction, and help to avoid and mitigate potential accidents that can happen during the operational phase. The digital twin can be valuable in work efficiency, system quality and safety management for construction, maintenance, and operational stages of the MRT.

## Literature review

The construction of railways has increased dramatically in recent years due to the rising density of people who live in the urban setting. Taipei City Government stated that the number of passengers traveling by public transportation was 30% in the Taipei metropolitan area in 2001, and by 2005, the mode share for railway had increased to almost 50% ^3^. Railway infrastructures can be formed by a range of structural assemblies, such as steel rails, sleepers, rail fasteners, concrete slabs, foundations, hydraulically bound, and/or ballast/sub-ballast layers. These assemblies are made from fundamental materials, such as steel, concrete, and rubber. The ballasted track is most-commonly used in traditional railway structures. Ballast bed is usually used to decrease the effective train loads to the ground, and it has an excellent performance in water drainage. The advantages of low consumption and simplicity of ballasted railway track maintenance significantly benefit railway developments ^4^, However, the frequency of maintenance works of the ballast layer and the associated vibration and noise causes inconvenience to residents, especially in urban areas ^5^. As a result, the consideration for installation of ballastless tracks gains significant momentum. The standard for ballastless tracks was first developed by the International Union of Railway (UIC) and British Rail’s Paved Concrete Track (PACT) ^6^. To optimize the quality of life in the city, Floating Slab Track (FST) is often used to meet the requirement of low-noise and low-vibration railways. A test result of the application FST in a light rail at Konya, Turkey demonstrated that the noise levels are about 1.6 times less than before the installation of FST ^7^. Non-Ballasted Track with High Isolation (NBHI) could also reduce the vibration by various isolation parameters through multilayers of isolation pads ^8^. For the case study in this study, FST and NBHI, which are installed from Chiang Kai-shek Memorial Hall Station to Dongmen Station, has been adopted for demonstrating the digital twin application to maintenance, risk, and resilience management.

### Maintenance of railway using BIM

Railway assembly is complex by nature. Risk management and maintenance is important so that any component failure, which could lead to hazardous accidents (such as derailment), does not occur. Moreover, the supply chain of materials and components is one of the key management issues for large-scale projects. It is very difficult to assign, schedule, and update material flows throughout the LCA using the traditional construction and asset management methods. To enhance the sustainability of the railway construction, a proper maintenance process is necessary. The design and management of the railway maintenance should consider all potential scenarios of the construction and operations. Three main factors contribute to defects that occur in railway tracks: (1) unexpected situations that occur during operation and maintenance, (2) surrounding environmental impacts, and (3) abrasion and erosion causing damage. In other words, exhaustive risk and hazard management in maintenance is needed through rigorous evaluation in multimeric parameters ^9^. Inaccuracies of information and records happen in the construction stage because the traditional risk assessment lacks up-to-date information, and uncertainties during the operation cause difficulties in comprehensive risk management ^10^. The adoption of BIM, which enables a multi-dimensional system, can improve risk identification in real-time. The various natural impacts such as flooding, seismic activity, changes in temperature, and geographic variation, can result in the shortened lifespan of the rail track ^11^. Earthquakes can cause instability in the foundations, especially in areas with soft soil. The risk of component failure during the operation stage could lead to a serious derailment situation ^12^. Evaluation of wear and tear of railway infrastructures is crucial to operational risk analysis. The risk factors, which are related to resistance reduction in rail infrastructures, should be robustly considered and frequently monitored in the design in order to improve safety and maintenance effectiveness of the railway system.

### Resilience

Maintenance is one of the most important activities in the operational stage over the life cycle of railway systems since detrimental accidents may occur if the track condition is deficient. The rapid development in the urban systems and built environments increases the complexity and interdependence of each mode of transport infrastructure, and this increases the difficulty in risk management among all assets ^13^. In the maintenance phase of the railway system, comprehensive risk analysis is required because it helps to improve sustainability and resilience. According to the European Technology Assessment Group (ETAG), various incidents that can happen regularly in a tunnel include fire, increased temperature, and flooding ^14^. These factors can have a serious impact on the railway system, leading to a service breakdown.

Taiwan is located on the circum-pacific seismic belt which results in a high frequency of earthquakes, approximately 40,000 per year ^15^. Earthquakes are also a threat to the railway system which would be affected by vibration or unexpected movement. The maintenance works in the metro railway system in Taiwan currently uses BIM to manage the working schedule, and the BIM is integrated with material inventory and a geographic information system (GIS) which analyzes the consequences that the underground geological structure has on the railway system ^15^.

To improve the quality and efficiency of the maintenance works, integrating all the inspection records with BIM tools can achieve optimum maintenance works. Visualization with a 3D model, coordination of a huge number of data, and powerful simulation in BIM can be applied to a railway system using AutoCAD Revit software ^16^, so that the maintenance can be managed in real-time.

### Knowledge gap

The application of BIM from the 1D to 6D approach has been widely used to support the construction of buildings and infrastructures in many projects ^17^. Some research using BIM analyses in different construction showed that the highest cost consumption is in the construction phase ^18^. However, there is still a lack of 6D BIM research and application to MRT construction and resilience management. Consequently, this study aims to fill the gap by demonstrating the extended BIM for Taiwan MRT that is capable of dealing with maintenance and resilience management over the life cycle. Depending on various environmental impacts, design parameters (such as radius of curvature which is affected by terrain and design speed) should be considered differently. Time consumption needed for construction and maintenance activities for different types of tracks is also a crucial factor to be embedded in the BIM. The human resource required in railway projects can depend on the amount of work and the size of the project, which can be implemented in the future extension of BIM.

## Methods

This study adopts Taiwan MRT as a case study to demonstrate new capabilities of digital twins in order to help planning and managing maintenance and resilience in practice. BIM of the Taiwan MRT has been developed using Revit, and multiple layers of datasets have been embedded in the BIM to generate the digital twin. In this study, Revit has been used to establish the BIM. After creating the 3D BIM model through Revit, detailed components and assembly can be verified and the bill of quantities can be determined. The model can then be imported into Navisworks. Navisworks v.2019 is used in this study to enhance the integration of the virtual model information (in Revit) and the maintenance schedule (e.g. by using multiple data layers and/or using data derived from other software such as MS excel spreadsheet). Similar packages are also embedded in alternative software such as Bentley BIM. After validation with field data obtained via the collaboration with Taiwan MRT (Track Engineering Department, MRT; personal communication August 2020), the digital twin has been used to demonstrate new insights on the sensitivity of material flow, hazard management, and lifecycle perspectives.

### Case study

**Figure 1.**
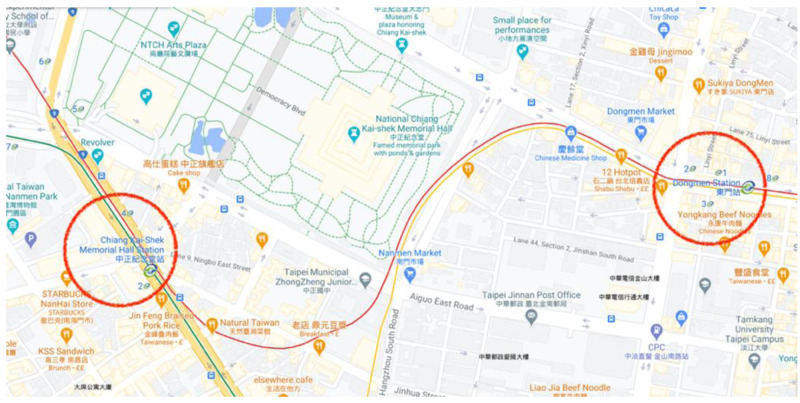
Track route from Chiang Kai-shek Memorial Hall Station to Dongmen Station (Red Line), Taipei, Taiwan (Courtesy: Google Map).

***The route.*** The track route of the Xinyi Line, which is known as the Red Line in Taiwan and has been chosen as a case study, is shown in Figure 1. Based on the project information of Xinyi Line (contract CK570H) (Track Engineering Department, MRT; personal communication August 2020), the length of the track between Chiang Kai-shek Memorial Hall Station and Dongmen Station is 1,219.3 m for upline and 1,225.8 m for downline. The track has been built in an underground tunnel and it is about 32 m in depth from the ground level. The route starts eastward at Chiang Kai-shek Memorial Hall Station, goes straight down Hangzhou South Road, turns east, and then goes down the Xinyi Road to arrive at Dongmen Station. The section comprises rail steel, electrical conductor rails, 24 assemblies at the end of power rails, eight insulated rail joints, four turnouts, a floating slab, and a non-ballasted track with high isolation.

***Utilization of track steel.*** The steel rail type is UIC 60, which is manufactured according to the European Standard EN 13674-1 ^19^. The UIC 60 rail steel is adapted to the Xinyi line with a standard track gauge. The route passes through the highly populated Taipei metropolitan area. Reduction of vibration during operation is important to ensure a good quality of life is maintained. Floating slab (FST) and non-ballasted track with high isolation (NBHI) have been chosen for the Taipei MRT railway track construction. The total upline length of FST (Red section) and NBHI (Green section) is about 420 m and 866 m, respectively; the total downline length of FST and NBHI is about 400 m and 881 m, respectively.

### Virtual modelling and maintenance assessment

***Estimation of Greenhouse Gas Emissions (GHG).*** Reduction of CO _2_ and energy consumption is a significant concern to the green construction in value engineering, which means that prediction of GHG emissions is required via the whole LCA. British Standards Institution (BSI) proposed ISO 14064-1 in 2006 and has widely implemented the standards ^20^. ISO 14064-1, which is termed an international standard for calculating the total carbon footprint emissions during the production of a product, can be calculated using Equation (1). 


GHGemssion=∑(I×f×GWP)(1)

Where *I* is the activities data, *f* is the parameter of GHG emissions and *GWP* is the coefficient of global warming potential which is given by the IPCC2007 report ^21^. The *GWP* value of carbon dioxide is 1. The total carbon footprint is computed for all the stages of the entire LCA. Table 1 and Table 2 presents the coefficient factors, which are given by the Bureau of Energy, Ministry of Economic Affairs (BOE) ^22^. The computation of carbon emissions in the operation and maintenance of railways is based on the activities of the maintenance works and carbon emission from the power consumption of facilities, such as offices or maintenance sites. The GHG emission associated with materials is mostly in the manufacturing and processing stage. Railway components, turnouts, and conductor rails are the three main components in the maintenance routine. When the amount of material and energy are known, they are used to calculate the amount of carbon emission by using GHG emission coefficients.

**Table 1.** Carbon emission factors ( **f**) by energy type in Taiwan ^25^.

Activity	Country	Unit	CO _2_e (kg CO _2_e)
Electricity Generated	TW	kwh	0.509
Diesel	TW	L	2.606
Fuel Oil	TW	L	3.111
Coal Fuel	TW	kg	2.535
LNG	TW	m ^3^	1.879

**Table 2.** GHG emissions factors per one unit of material ^25^.

Activity	Country	Unit	CO _2_e (kg CO _2_e)
Concrete (280 kgf/cm ^2^)	TW	m ^3^	162.74
Cement	TW	kg	0.95
Steel bar	TW	kg	2.63
Rebar	JP	kg	2.09

**Figure 2.**
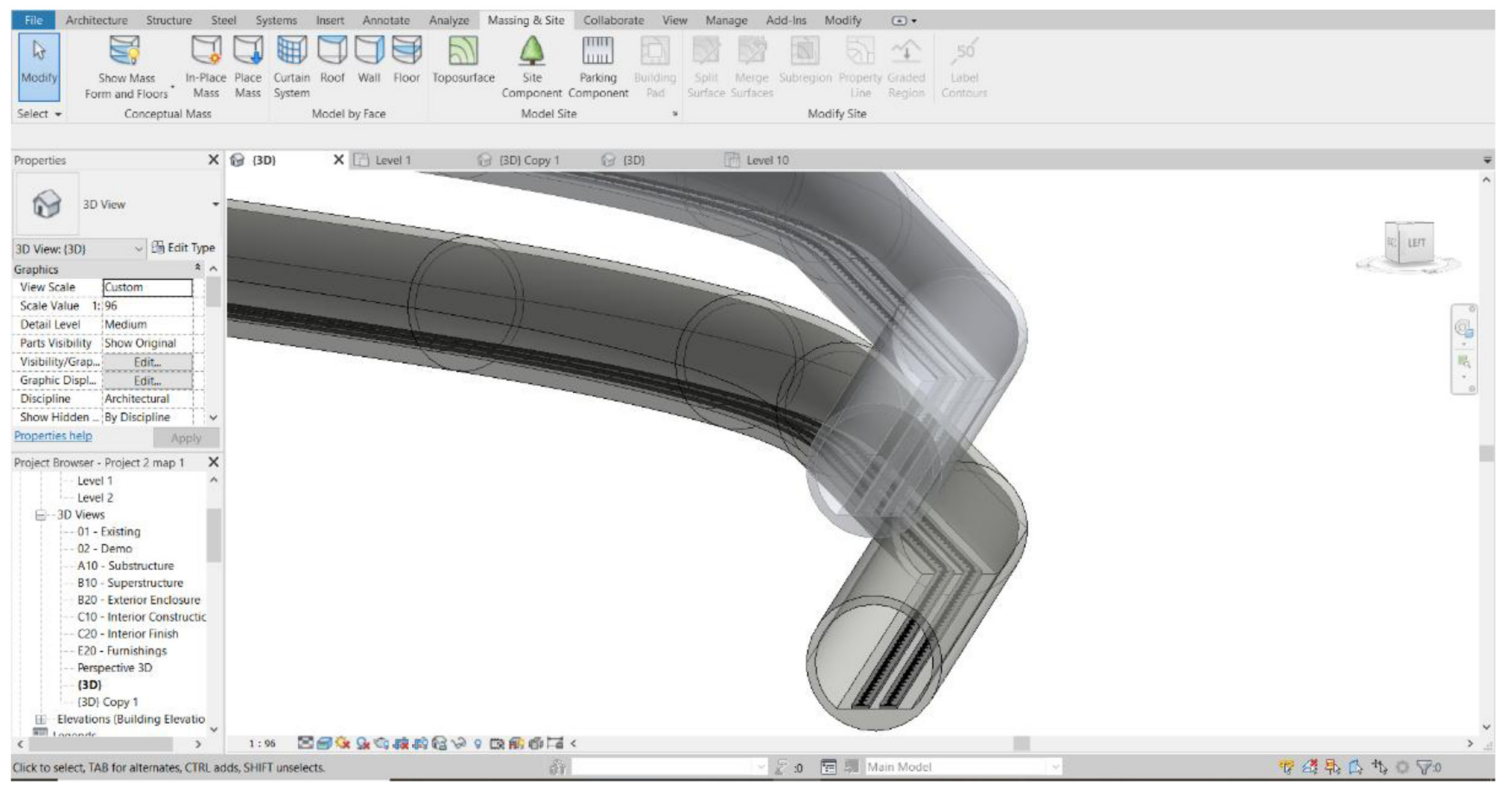
3D model of MRT metro developed using AutoCAD Revit in this study.

***3D Modelling.*** Figure 2 demonstrates the 3D model developed from the fundamental information and 2D drawings of the MRT track infrastructures ^23, 24^. We have used the free version of AutoCAD Revit 2020.2 to develop the 3D model of built environments, which is available to students, teachers, and educational institutions. Free, open source alternatives to AutoCAD Revit that can be used to replicate this study are FreeCAD, BIMSpot (which allows every stakeholder to work on a single BIM model, and SketchUp. As auxiliary BIM tools, the 3D structure can be modified in real-time progress, and the conflicts between components (and data sets) can be detected in the clash detection feature. BIM has been used to analyze the material inventory and the flow throughout LCA. The main materials utilized in the railway construction are concrete, steel, and natural rubber, and the environmental risks to the railway construction have been determined using the schedule function that is included in the software. The material management (via BIM volumetric quantification) has been arranged by the bill of quantities, and the detailed information such as dimensions of components; and itemized costs have been input into BIM to achieve comprehensive integration.

### Sustainable maintenance and optimization

The frequency of track inspections is one of the critical factors influencing the quality and reliability of the railway infrastructures, since safety is more likely to be compromised if the period between maintenance inspections is much longer. Nevertheless, if the maintenance period is too short, this can increase costs and reduce time efficiency. The railway systems maintenance work has been clarified into two phases: corrective maintenance (CM), and preventative maintenance (PM). When damage has occurred in the construction or during the asset operation, the emergency repair needs to be done immediately, and so it is classified as break-down or CM. If the deterioration is found in construction or operational stages during the track inspection, and the aging parts are repaired or improved before the full damages or failures occur, it is termed PM. The MRT system is designed to be a long-term service transportation in the metropolitan area, and so it is important to ascertain its resistance, safety and sustainability by effective maintenance that an appropriate maintenance schedule is required. PM is considered to be more desirable for the sustainability of the rail infrastructures since it gains more financial benefits than corrective maintenance. A comprehensive PM requires a suitable condition-based monitoring system to determine and prioritize risk profiles and the engineering team can promptly respond through adaptive maintenance works. In this study, the life cycle assessments take into account the maintenance schedule, costing and carbon emission in accordance with the preventive maintenance concept.

***Frequency of maintenance and cost estimation.*** Table 3 demonstrates the detailed activities of the preventive maintenance in MRT systems. The detailed activities are obtained through communication with Taipei MRT (Track Engineering Department, MRT; personal communication August 2020). The inspection in the schedule is planned to prevent any deteriorations and to check the conditions of the assets and facilities. The maintenance schedule could be slightly different between track sections depending on the service condition (e.g. axle load, train speed, etc.) and track parameters (such as curves and geometries, etc.) in reality.

**Table 3.** Heavy Rail Preventive Maintenance Schedule in MRT systems ^24^.

Item	Ref. no	Activity	Frequency	Material Cost (£)
Railway Components	1	Track and conductor rail systems maintenance by patrolling along the mainline.	Weekly	6.63
	2	Track alignment and rail steel abrasion inspection	Every 2 months	80.06
	3	Thermite welding rail joints (non-destructive inspection)	Every 2 months	93.87
	4	Insulated joint inspection	Every 6 months	11.26
	-	Buffers stop inspection	X	X
Turnout	5	Gap between ball and claw inspection for mainline	Monthly	5.87
	6	Crossing nose, switchblade, and associated devices inspection for mainline	Every 6 months	15.81
Conductor rail	7	Clearance	Every 3 months	53.49
	8	Rail joints and abrasion rate inspection	Annually	9.62
	9	Support components and insulation value inspection	Annually	0.73
	10	Abrasion rate and elevation position inspection (Standard track inspection vehicle)	Every 6 months	0.73
FST Slab Track	11	Slab track inspection (include repair and replacement)	Every 2 years	21.96

The material cost has been determined in relation to the maintenance activities tabulated in Table 3. The time consumption has been determined by expenditure hours in a one-time inspection, and the aggregate working hours are equal to the unit time consumption multiplied by times for the relative events as shown in Equation (2) and Equation (3). In this study, the total time and material cost per year are used in the calculation of life cycle cost estimation, which is corresponded to the length of the route, material, and used assets and facilities.


UnitMaterialCost(£)×Numberoftimes=TotalCostConsumption(£)(2)

UnitTimeconsumption(hour)×Numberoftimes=Aggregateworkinghours(3)

***Material usage.*** The material stock is important to the entire project, and appropriate management to monitor the material flows is vitally needed. Table 4 represents the material list for the components in MRT systems. The life cycle analysis is based on the whole-life activities of scheduled maintenance works. Note that common damages occurring in the material includes rail steel abrasion (i.e. wear and tear), rail surface damages (e.g. squats, corrugation, rolling contact fatigue, shelling, etc.), concrete carbonation, steel corrosion, and cracks on the surface of concrete beds. The material inventory is monitored by the Revit software which is used in the study so that engineers can check the requirement rapidly via the digital twin (DT). During the inspection, the flow of the material via the LCA and damages can be analyzed using BIM. The materials used in the maintenance can also be considered in the calculation of the amount of carbon emission by using GHG emission coefficients.

**Table 4.** Material list for track used in the studied line ^24^.

Component	Item	Material
Track	Slab Track	Concrete/ frameworks
	UIC 60	Stainless steel
	Elastomeric Bearing	Butadiene rubber
Conductor rail	Rail pad	Natural Rubber/ Ethylene Vinyl Acetate Copolymer/High-Density Polyethylene
	Clip insulator	Natural Rubber/ Ethylene Vinyl Acetate Copolymer/High-Density Polyethylene
	Rail clip	Steel
	Anchor assemblies	Galvanized steel
Fastening system	Conductor rail	High conductivity steel
	Insulation Components	Galvanized steel
	Splice-Joint Assembly	Steel
	Expansion-joint assemblies	Stainless steel
	Anchor assemblies	Galvanized steel
	Power connectors and cable assemblies	-
	Cover	-

## Results

### Virtual modelling and maintenance assessment

***Estimation of Greenhouse Gas Emissions (GHG).*** The GHG emission of the material has been embedded in the manufacturing phase. By using the digital twin of Taipei MRT track section, the quantity of concrete, steel, rebars and rubbers (including energy consumption in their production) can be determined for the calculation of GHG. The results exhibit that the quantity of concrete, steels and rebars is about 37% (2310.63 tCO _2_e), 22% (1374.24 tCO _2_e.) and 41% (2528.21 tCO _2_e.) respectively (see *Underlying data*).

The carbon emission during the operation and maintenance is computed by using the electricity consumption of the depot, and is approximately 23.8 kWh/car.km ^3^. The power is approximately 5,928,043.234 kWh/ year, multiplied by the GHG emission factor of electricity, the total carbon emission is approximately 3017.37 tCO _2_e (see *Underlying data*).

**Figure 3.**
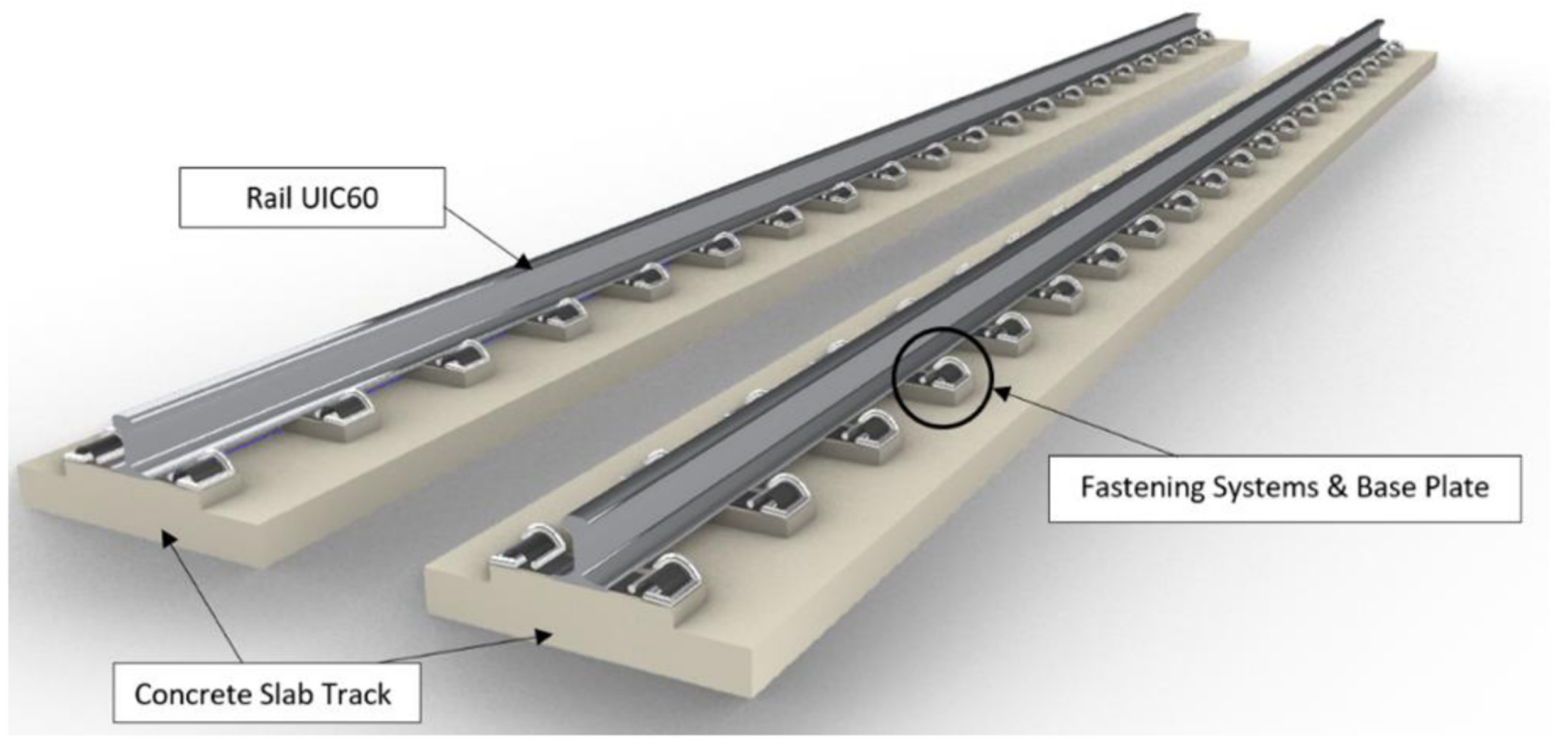
3D components of Non-Ballasted Track with High Isolation (NBHI) track.

**Figure 4.**
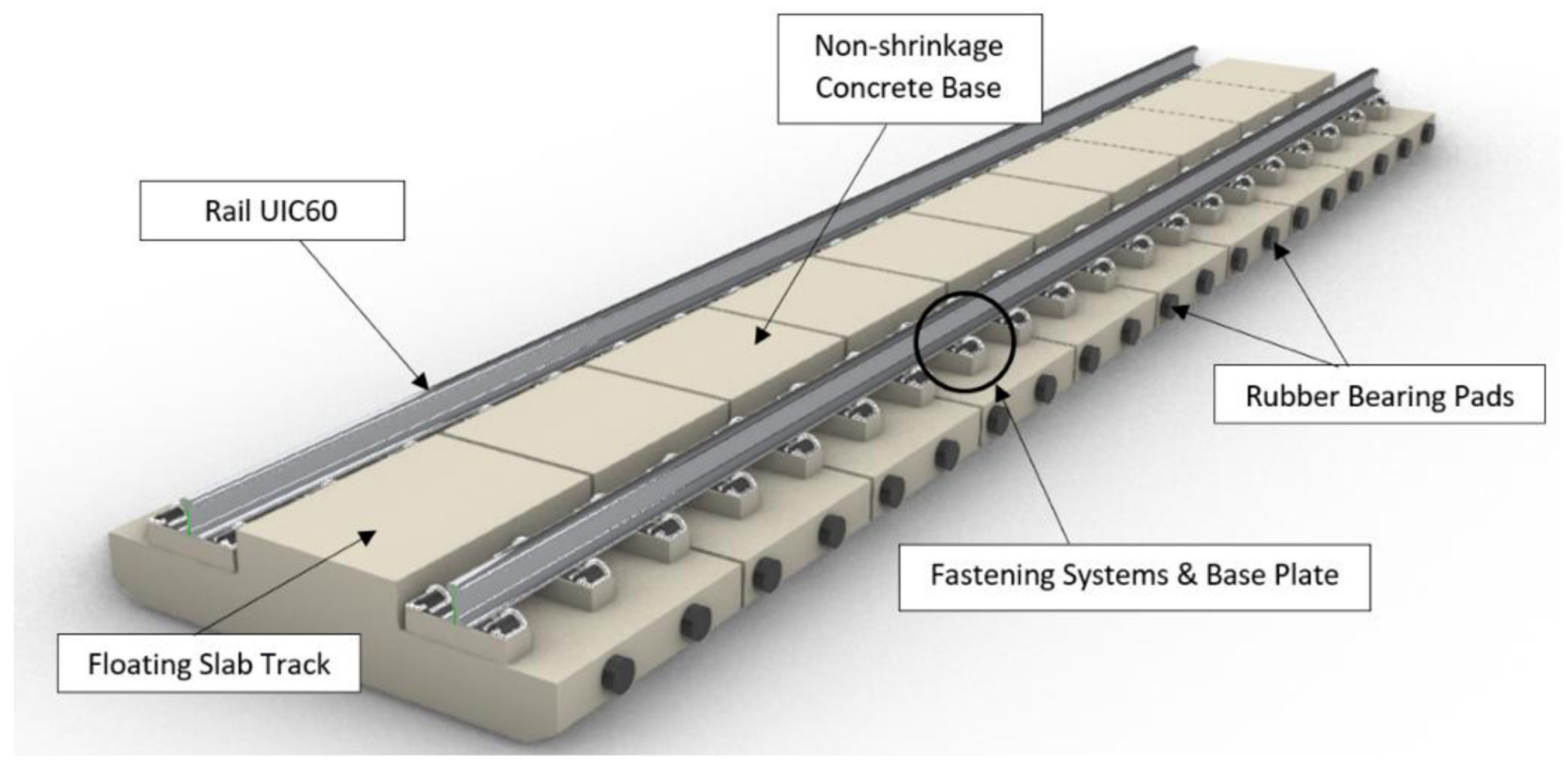
3D components of Floating Slab Track (FST).

**Figure 5.**
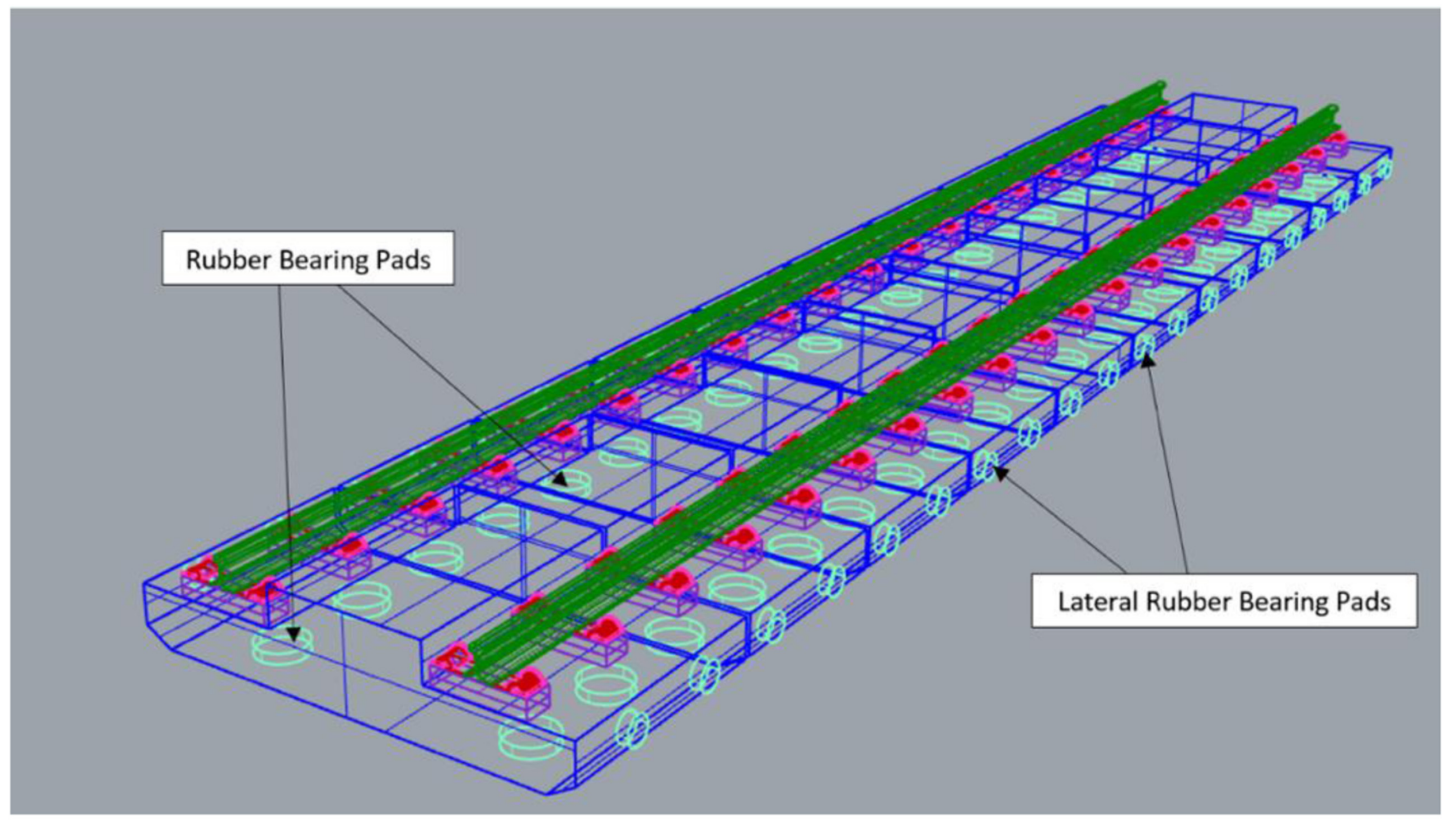
Position of the bearing pads of Floating Slab Track (FST).

***3D modeling of the track.*** The 3D model of Taipei MRT track section has been established in collaboration with Track Engineering Division of Taipei MRT ^23, 24^. Figure 3 and Figure 4 visualise the detailed 3D components to the NBHI and FST tracks established for this case study. As seen in Table 4 ^24^, the main components to construct a ballastless railway track are rail steel, concrete track slab, fastening systems, and track support systems. FST slab beds, which are made by steel rebar frameworks and concrete, are manufactured off-site. According to a report published by the Department of MRT ^24^, the weight of the framework is about 2.5 to 3.5t, and the standard dimension of a track slab is 2510 mm × 1450 mm × 280 mm. The bed has 4 circular indentations, and that space is reserved for the bearing pads underneath the slab ( Figure 5). The lateral bearing pads are installed at the two sides of the track. In the calculations, the values of the standard design and average values (such as dimensions, weights, and components of rail components) have been applied. Depending on the different causes that lead to damages, the maintenance method can vary, since inappropriate works could lead to critical damages or failure of the system.

**Figure 6.**
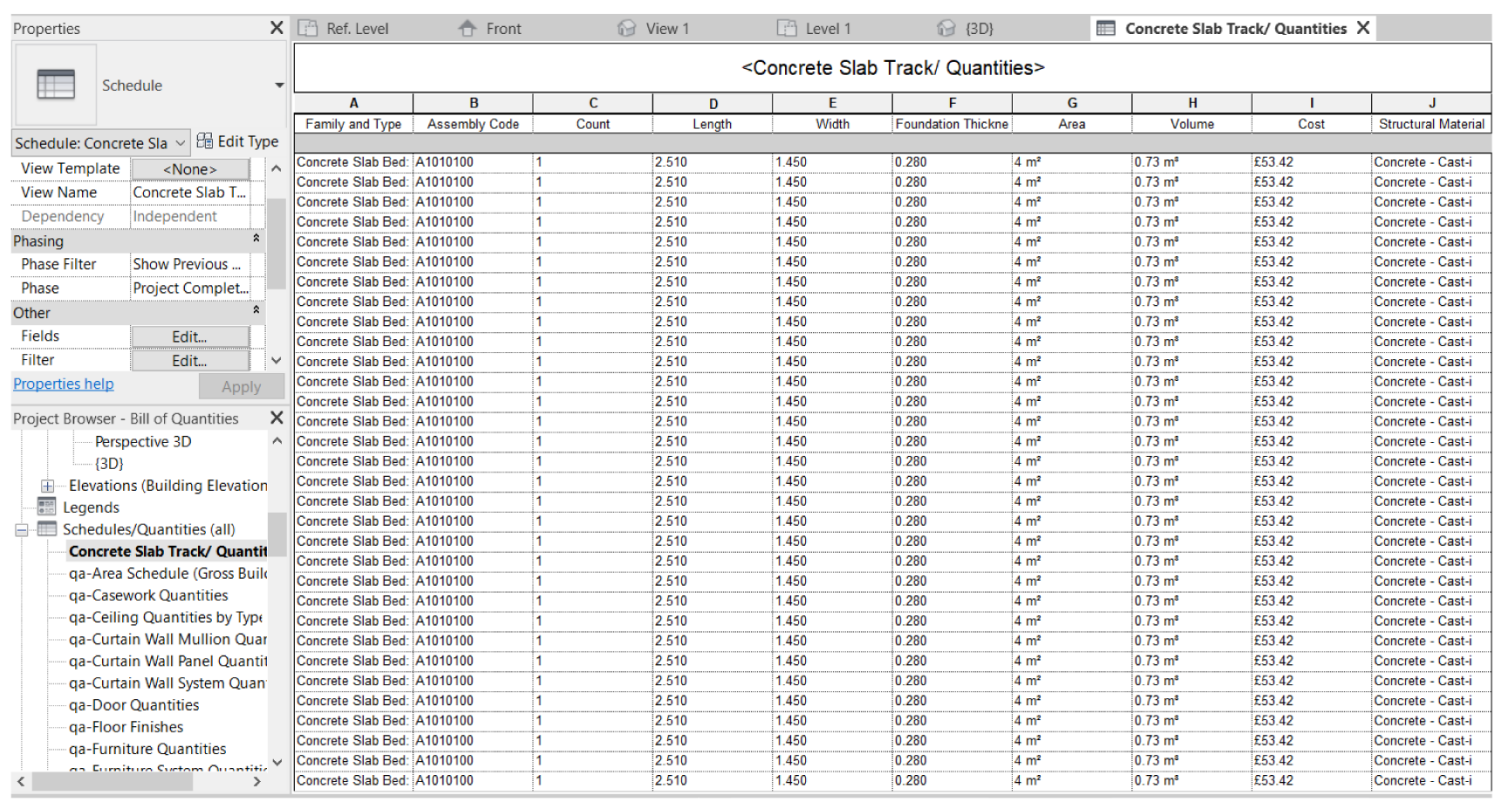
Bill of quantities list of concrete slab track.

***Bill of quantities.*** The railway track in the MRT system consists of a large number of complex components, and the traditional inventory method in practice lacks efficiency. Mining historical data records takes a long time and the existing approach has issues of losing detailed information or lacking of documents integration. Figure 6 demonstrates the bill of quantities of the concrete slab beds of the FST track section, quantified using the established digital twin (i.e. Revit). The list can be updated to reflect variations in cost and requirement of maintenance activities. The digital twin brings significant benefits to all stakeholders who are involved in the project. It is applicable to all stages of the railway project through the whole life cycle, and it has a remarkable influence on maintenance and resilience management in the operation and maintenance phase especially. In the MRT system, maintenance is largely impacted by various factors in different life cycle stages. Proper methods and task arrangements are thus important during the maintenance. Good organization is greatly important to time and cost efficiency, and can decrease the project risks. Generally, the goal of preventative maintenance is to optimize the resource allocation that can remove the potential for damage in time prior to causing a severe danger. To effectively enable the preventative maintenance regimes, the bill of quantities in Revit can be used to monitor the inventory and the flow of the material used at any time.

Material stocks management, which integrates detailed information such as budgets, transportation and planning, is important to the project. The material price fluctuation has a big influence on the project budget, and the managers can achieve the optimal purchasing prices by comprehensive material management.

### Sustainable maintenance and optimization

***Frequency of maintenance and cost estimation.*** The cost estimation is done based on Equation (2). For example, the cost consumption in insulated joint inspection is £11.26 every time, and the task or event is executed every 6 months so that the inspection for this event will be held twice per year. The aggregate working hours is computed by Equation (3). If the time expenditure of insulated joint inspection is 2 hours in one time, and the task is executed every 6 months so that the inspection for this event will be held twice per year.

The inspection of track and conductor rail has higher expenditure than others (40%). The weekly inspection is to ensure that the facilities meet the safety standard, enabling predictive and preventative maintenance concept. The three main costs in the inspection are thermite welding rail, track alignment abrasion checking, and track and conductor maintenance which are about £563.25 (32%), £480.35 (27%), and £351.36 (20%), respectively.

**Figure 7.**
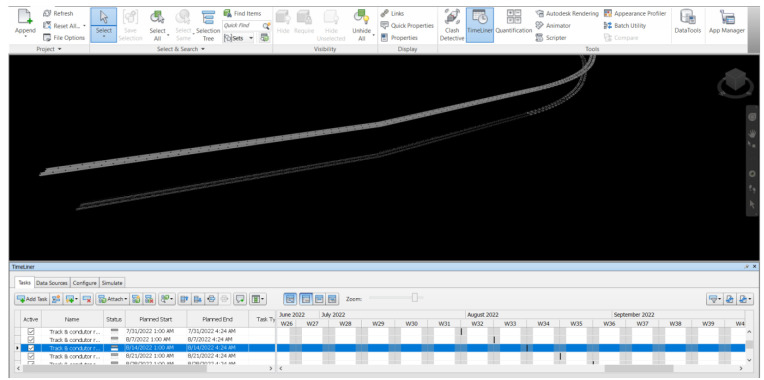
Integration of 3D model and maintenance schedule by Navisworks 2019.

***Maintenance schedule and 3D model.*** Figure 7 demonstrates the maintenance schedule information integrated with the virtual model by using Navisworks v.2019 developed in this study. After creating the 3D model and obtaining the bill of quantities from Revit, the model has been imported into the Navisworks. Navisworks v.2019 is used to analyze the integration of the virtual model information and the maintenance schedule with the timeline in the software to keep the information in BIM up to date. The material stock will be updated from the condition of the most-recent maintenance. The insight can help engineers for better and more effective material management, which can eliminate waste for excessive inventory.

## Discussion

### GHG emission/cost/ total time consumption

Based on the results, it is very clear that, among all of the track components, the majority of GHG emission is derived from the track slabs. This is because track slabs have to redistribute and absorb high-intensity dynamic forces from the train prior to transferring to the track support and tunnel floor. On this ground, the track slabs have been conservatively designed, and then their resultant dimension and volume become overly large, of which the track slabs contribute to much higher consumption of materials (such as concrete and reinforcement), excessive cost and GHG emission. This implies that the next generation of track slabs requires better design and optimization toward net zero GHG emission. This aspect indicates a new field of transdisciplinary research into more sustainable track slab systems.

Surprisingly, the results based on Taipei MRT case study exhibit that, when considering throughout the life cycle, the construction phase of track infrastructures contributes the most GHG emission compared with train operation and asset maintenance phases. This is because Taipei MRT has already exercised green energy grids where the power and electricity used in railway network (for train tractions, for railway stations, for track infrastructures, for signaling and control) are relatively clean (e.g. from solar power) and do not emit significant GHG. This demonstrates a new paradigm for other railway networks globally to learn from.

When considering the risks and resilience in asset management, it is found that more frequent and robust track inspection regimes are required when the infrastructure are exposed to uncertain settings such as natura hazards and so on. The case study of real-life MRT has clearly demonstrated that the use of digital twin can significantly improve the efficiency and effectiveness of maintenance responses when defects or damages occur. This is because the digital twin can help (i) locate the defect accurately; (ii) promptly share and visualise information needed by related stakeholders in order to make decision and act on the priority; (iii) create rapid and virtual collaboration that synergizes various expertise from all stakeholders; (iv) rapidly interact with on-side sensors and on-board condition monitoring systems that determine most up-to-date system integrity; (v) link with material stocks to determine the logistics and supply chain capacity of components and materials for repair and/or maintenance actions; and (vi) help engineers, maintainers and technicians to determine lifecyclce costs, GHG in order to make a decision that can lead to a more sustainable construction and maintenance of railway infrastructures.

### Replacement and repairs

After inputting maintenance records into Revit and Navisworks, and processing them via the schedule function that is included in Revit, examples of the replace and repair activities can be compiled (as shown in Table 5). Note that multiple dimensions of other data sets (such as risks and vulnerability, hazards, sensor location, maintenance priority, base operating conditions, required activities, etc.) can also be appended in the schedule layer of the project.

**Table 5.** List of maintenance activities.

Causes	Actions
Loose bolts or clips in fastening systems	• Adjust the parts and check if the fastening system is corresponding to the standard. • If the fastening systems are unacceptable after testing, they need to be replaced immediately.
Base plates get serious corrosion, broken or displacement	• The failure parts should be replaced immediately and check the impact factors.
Aging phenomenon or damage occur inelastic materials	• The elastic material should be replaced if it is broken down, and the aging material should be repaired to the allowable safety value.
Cracks occur in fixed components or bolted concrete	• Determine the contaminants leading to cracking, such as chlorides, carbon dioxide sulfates, etc. • Use epoxy resin or polyurethane material to seal cracks to prevent the damage from deterioration of erosion.

### Potential damage in materials

The main materials that form the components usually take the biggest impact from damage. In practice, defects appear in aging materials and components after long-term operation. Aided by the DT database developed in this study, engineers can take actions based on the historical maintenance records and on-site conditions (obtained from either inspections or condition monitoring sensors) during the maintenance phase. According to differential damage conditions, the repair methods and maintenance actions can be different, and the required materials can vary during the maintenance phase (Track Engineering Department, MRT; personal communication August 2020). The following section presents the causes of damage and the methods to repair such damage found during the inspection ^23, 24^.

(1) Concrete
  • For the interior attack, the corrosion can result in cracks on concrete when the active anode in the steel bars increase. For the shallow cracks, it can be filled with epoxy mortar after clear fragments and dust stuck in the cracks. If the crack exceeds the acceptance, the parts should be replaced.
  • Carbonation is one of the main problems for the concrete since it can deteriorate the corrosion of rebar inside. Epoxy coatings and silane-siloxane sealer cover on the surface of the concrete can decrease the electrochemical reaction with water and CO _2_.
(2) Metal/ Steel/ Aluminum alloy
  • The risk of corrosion occurs due to concrete carbonation. The reinforcement inside the concrete is corrosively active especially when exposed to moisture. To stop the chloride contamination from aggressing further, coating the surface of the concrete can pause the degradation; however, the corrosion of the steel bars can be too severe, it should be replaced immediately.
  • For steel components with little corrosion, appropriate painting could be applied as a protection layer to stop oxidation (reaction between steel and with oxygen after removing the rust part).
  • The steel rail should be replaced depending on the rate of abrasion (wear and tear), fracture, and fatigue failure; if the rail is not within the acceptable wear rate (i.e. abrasion level representing by the percentage of cross-sectional area loss), the rail should be replaced immediately.
(3) Rubber
  • The embrittlement and cracks can occur in the aging rubber and fastener due to residual moisture and ambient heat. Engineers and technicians need to assess the stiffness and damage condition of the elastomers and fasteners, if no cracks appear, the greases, dirt, water, etc should be removed to restore the parts. If cracks occur, the component should be replaced immediately.

According to the different maintenance requirements of components in the MRT construction ^24^, the flow of material influences the cost consumption and the efficiency of the workflow in the project.

### Environmental impacts

Since the CK570H section is underground, the track is barely affected by weather conditions such as wind, rainfall, etc. However, some potential environmental impacts still need to be considered to avoid any unexpected situation causing damages or further risk to the infrastructures.

(1) Flood

Although most of MRT track system is the underground section, flooding can occur if there is a heavy rainfall exceeding the drainage capacity in the area. Generally, the annual average rainfall precipitation over the world is about 100 cm ^26^. During 1991 – 2020, the average regional rainfall precipitation in Taipei was 236.85 cm ^26^. During the summer, typhoons can still produce significant precipitation such that the flooding leads to a breakdown of the MRT system. Another indirect disaster that causes flooding is an earthquake, the active fault would lead to the displacement of the underground. The breakage of the water pipes and underwater leaking from the cracks can result in serious flooding in the tunnel. The methods to avoid further damages to the railway system is presented below ^23, 25^:

• Installation of full-section floodgates in the underground tunnel is required. The system is then monitored and controlled by operators in the central control room (CCR). When the flooding accidents occur, the emergency alarm will rise if the water level is over the acceptable capacity, and the floodgate would be closed to prevent the MRT systems from further damages.
• The damage from the water ingress attacks the concrete, which is the key factor leading to material failure due to water contaminates. From the exterior side, a waterproof membrane, which is a cover on the concrete foundation, could be a water-resistant layer. From the interior side, the ingredients of the concrete could be adjusted to be waterproof concrete to improve the strength of the structure. However, they are the temporary protection to decrease the harms from this disaster; thus, the detailed inspections must be conducted after the flood.
• Since Taiwan is located on the seismic belt distribution, a high frequency of earthquakes occurs. The seismic monitoring system is used to collect the seismic data, and it is estimated that there are about 20,000 earthquakes s on average annually. Among all earthquakes, around 1,000 times is a classed as a feeble earthquake ^27^. Not only could the earthquake indirectly lead to flood, but It could also cause damages to the MRT systems. To avoid any detrimental accidents, the following actions should be taken if the earthquake occurs ^23, 25^:
(2) Earthquake
  - The earthquake could be monitored and controlled in the CCR by the environmental control system. The sensors, which are installed along the tracks and on-board vehicles, can send the information immediately to the control system. All the systems could be stopped when the alarm is raised in case of any further consequences (such as aftershocks). The seismic warning is categorized into 3 levels depending on the situation, and the CCR could determine whether it is appropriate to resume the operation.
  - The displacement due to an earthquake could damage railway facilities. The inspection of track irregularity should be executed to ensure that geometric parameters of rails are within the tolerance range. If the parameters exceed the acceptance, it needs to be repaired immediately.

## Conclusion

In practice, railway construction is a mega project that usually involves many stakeholders and companies who can benefit from data integration and sharing. The lack of effectiveness in asset management may lead to serious accidents, especially in the operation and maintenance stage. Digital twin which acts as a powerful information platform (through the integration of BIM and Navisworks), can help to solve these problems. Not only can the digital twin contain a pool of information, but it also enables access to information throughout the whole life cycle of a project. By using the digital twin, all stakeholders relevant to the project can collaborate, co-create, and collect the latest progress in real-time. The database within the digital twin can help to achieve the sustainability goal by enabling efficient asset management and monitoring, and it could determine and control the carbon emission, cost estimation, and time management throughtout the whole life-cycle.

This study aims to demonstrate how a digital twin (integration of BIM and Navisworks) achieves project sustainability by the information integration in railway construction. Taipei MRT system is one of the main public transportations in Taiwan; and its construction has expanded the development growth of the metropolitan. The case study has demonstrated the ability of the digital twin to implement life cycle assessment, and revealed that the material inventory can be monitored by an interactive BIM model using Revit or equivalent (e.g. FreeCAD, BIMSpot, SketchUp, or Bentley BIM). The case study has also demonstrated that the repair works can be executed smoothly through the digital twin during the maintenance stage. According to the different conditions in various parts/elements of the construction, the proper repair methods should be taken into account in the system design. It is apparent that Navisworks can help to monitor the schedule execution in real time. Its interaction with BIM can help engineers and infrastructure managers to efficiently arrange the flow of material stocks and effectively respond to the material demand base on the situations considering risks, vulnerability, and maintenance requirements.

This study exhibits that digital twin is a technology that benefits project management, condition monitoring, and asset operation throughout the entire life cycle in real time. In the case study, BIM acts as an integrated database that contains the information on the design, construction, operation, maintenance, and decommissioning phases. The quality, efficacy and effectiveness of operation and maintenance phase are then deeply correlated with the life-cycle assessments. In this study, the workflow and inventory of the material stocks are embedded in the BIM so that the repair methods and maintenance actions can be optimised to decrease the track component and material deterioration. It should be noted that a number of potential risks such as environmental factors can affect the track inspection plan. The integrity of materials and components is critical to operational safety and reliability of Taipei MRT network. Improper asset and maintenance management will lead to unplanned maintenance resulting in excessive cost and time, and potentially a delay of any maintenance task. On this ground, this study unprecedentedly demonstrates the use of digital twin to tackle sustainability and resilience challenges in railway asset management throughout the life cycle under uncertainties (derived from various natural hazards). It is noteworthy that this study has already resulted in a new guideline for digital twin adoption in Taipei MRT network for lifecycle asset management and resilience monitoring. The insights from this study can also help engineers, asset managers, infrastructure maintainers and other stakeholders to collaborate and co-create sustainability values for the railway industry globally. 

## Data availability

### Underlying data

Zenodo: Carbon emission and lifecycle costs supporting digital twins for managing railway maintenance and resilience. https://doi.org/10.5281/zenodo.5034476 ^28^.

This project contains the following underlying data:

- Project Calculation.xlsx. (material used in a railway project, detail of railway maintenance, and carbon emission calculation)

Data are available under the terms of the Creative Commons Attribution 4.0 International license (CC-BY 4.0).
