# Supplementary material for: Digital twins for managing railway maintenance and resilience
Source: Open Res Eur. 2021 Nov 1;1:91. Originally published 2021 Aug 4. [Version 2] doi: 10.12688/openreseurope.13806.2 (PMC10445889; doi:10.12688/openreseurope.13806.2)
Supplement: Peer review history

# Peer review: Reviewer response for version 1

**DOI:** 10.21956/openreseurope.14885.r27916

**Published:** 2021-10-27

**Authors:** Yunlong Guo

**Affiliations:** 1 Faculty of Civil Engineering and Geosciences, Delft University of Technology, Delft, The Netherlands

In general, this is a very good paper. It presents a study case of using BIM for Metro LCA. The interesting part is the paper stressed the GHG emissions. Only a few small viewpoints need justification. For example, the noise and vibration of ballasted track disturb residents may not be very precise. Because the noise of slab track in metro is also very big. The noise is mainly from the train braking and starting, especially the parts with rail corrugation.

 The prediction of CO2 can be very precise, however, predicting the maintenance is quite challenging. It seems the maintenance records of the metro line are not shown in the paper. Maybe, inputting some real maintenance, especially the corrective maintenance. Because it is easy to use the data of PM for analysis, however, including CM will make the study with more accurate prediction.

Is the study design appropriate and does the work have academic merit?

Yes

Is the work clearly and accurately presented and does it cite the current literature?

Yes

If applicable, is the statistical analysis and its interpretation appropriate?

Yes

Are all the source data underlying the results available to ensure full reproducibility?

Yes

Are the conclusions drawn adequately supported by the results?

Yes

Are sufficient details of methods and analysis provided to allow replication by others?

Yes

Reviewer Expertise:

railway engineering. railway ballast. DEM. Image analysis

I confirm that I have read this submission and believe that I have an appropriate level of expertise to confirm that it is of an acceptable scientific standard.

# Response

**Published:** 2021-10-28

**Authors:** Sakdirat Kaewunruen

**Affiliations:** University of Birmingham, UK

The authors wish to thank the reviewer for the valuable comments and commendation. The CO2 data has been archived in European open data platform via https://doi.org/10.5281/zenodo.5034476.

 Note that the real maintenance data has been adopted throughout this study as part of the collaborative project with Taipei MRT. In this paper, the context of noise and vibration (N&V) is only related to the N&V due to construction and maintenance activities. The use of slab track can yield lesser maintenance activities, which will help improve this issue.

# Peer review: Reviewer response for version 1

**DOI:** 10.21956/openreseurope.14885.r27389

**Published:** 2021-08-26

**Authors:** Sreevalsa Kolathayar

**Affiliations:** 1 National Institute of Technology Karnataka, Surathkal, Karnataka, India

The paper is well written and can be accepted for indexing. It presents a case study where track 3D modeling is employed scientifically. The method suggested could be useful in more efficient management of track maintenance. The resilience aspects are also discussed in context of flood and earthquake. However the strategies suggested towards resilience against floods appear to be generic. It could be made more specific in terms of results obtained from BIM.

 The conclusions can be more focused and specific.

Is the study design appropriate and does the work have academic merit?

Yes

Is the work clearly and accurately presented and does it cite the current literature?

Yes

If applicable, is the statistical analysis and its interpretation appropriate?

Yes

Are all the source data underlying the results available to ensure full reproducibility?

No source data required

Are the conclusions drawn adequately supported by the results?

Yes

Are sufficient details of methods and analysis provided to allow replication by others?

Yes

Reviewer Expertise:

Geotechnical Engineering, Earthquakes, DRR

I confirm that I have read this submission and believe that I have an appropriate level of expertise to confirm that it is of an acceptable scientific standard.

# Response

**Published:** 2021-09-01

**Authors:** Sakdirat Kaewunruen

**Affiliations:** University of Birmingham, UK

The authors wish to thank the reviewer for the valuable and highly constructive comments, which can help significantly improve the quality of the work. All review comments have been adopted in the revision and have been fulfilled in the following details:

- More details have added to the methodology part.
- Flood management aspect has been further discussed in the discussion part.
- More details have been appended in the result part.

# Response

**Published:** 2021-10-28

**Authors:** Sakdirat Kaewunruen

**Affiliations:** University of Birmingham, UK

The authors wish to thank the reviewer for the valuable comments, which can significantly improve the quality of the work. The comments were fulfilled in the following details:

- More detail was added to the methodology part.
- Flood management was more discussed in the discussion.
- More detail was included in the result part.
